# Determinants of internal medicine residents' choice in the canadian R4 Fellowship Match: A qualitative study

**DOI:** 10.1186/1472-6920-11-44

**Published:** 2011-06-29

**Authors:** Vijay J Daniels, Narmin Kassam

**Affiliations:** 1 Department of Medicine, University of Alberta, 2F1.13 WMC 8440 - 112 Street, Edmonton, T6G 2B7, Canada

## Abstract

**Background:**

There is currently a discrepancy between Internal Medicine residents' decisions in the Canadian subspecialty fellowship match (known as the R4 match) and societal need. Some studies have been published examining factors that influence career choices. However, these were either demographic factors or factors pre-determined by the authors' opinion as possibly being important to incorporate into a survey.

**Methods:**

A qualitative study was undertaken to identify factors that determine the residents choice in the subspecialty (R4) fellowship match using focus group discussions involving third and fourth year internal medicine residents

**Results:**

Based on content analysis of the discussion data, we identified five themes:

1) Practice environment including acuity of practice, ability to do procedures, lifestyle, job prospects and income

2) Exposure in rotations and to role models

3) Interest in subspecialty's patient population and common diseases

4) Prestige and respect of subspecialty

5) Fellowship training environment including fellowship program resources and length of training

**Conclusions:**

There are a variety of factors that contribute to Internal Medicine residents' fellowship choice in Canada, many of which have been identified in previous survey studies. However, we found additional factors such as the resources available in a fellowship program, the prestige and respect of a subspecialty/career, and the recent trend towards a two-year General Internal Medicine fellowship in our country.

## Background

There is a discrepancy between Internal Medicine residents' career choice in the subspecialties and societal need [1]. Specifically, there has been a declining interest in General Internal Medicine since 1998. Fifty-four percent of American third year Internal Medicine residents planned to practice General Internal Medicine in 1998 [2]. This number drastically declined to 27% of third year residents and only 19% of first year residents planning to pursue careers in General Internal Medicine in 2003.

A similar problem exists in Canada where the number of Internal Medicine residents pursuing General Internal Medicine is similar to that of the United States. Based on CAPER (Canadian Post-MD Education Registry) data between 2004-2008, 18-23% of third year Internal Medicine residents pursued General Internal Medicine as a fellowship in the R4 subspecialty match (i.e. after their core three years of Internal Medicine) [3]. Canadian physician resource studies predict a critical shortage of generalists over the next five years given that 300 to 500 general internists will retire with only half being replaced by new graduates [4]. This concern regarding career choice and manpower in General Internal Medicine is not just localized to North America [5-8] and is in fact a global problem.

The reasons why residents choose their fellowship are not completely clear. Some survey studies have been published examining factors that influence career choices in Internal Medicine [1,2,9-13]. One particular issue with these studies is that the factors investigated are either demographic or pre-determined by the authors' opinions as possibly being important to incorporate into a survey. Also, these studies almost exclusively involve American residents only. One study by Horn et al. [11] did focus on Canadian residents and had a qualitative component to further evaluate factors identified in a previous survey of the participants. However, we are not aware of any purely qualitative studies that have used unprompted residents' opinions to establish important factors that influence Canadian Internal Medicine residents' choice in the R4 match.

## Methods

### Ethics

We obtained ethics approval for this project through our university's Health Research Ethics Board prior to commencement of recruitment and data collection and we obtained informed consent from all participants.

### Recruitment

In Canada, all Internal Medicine residents must complete three years of core Internal Medicine training and then they decide whether they are going to subspecialize (an additional two or three years of training) or continue in General Internal Medicine (mandatory one year, optional second year of training). This decision is made five months into the third year of core training. We wanted to include residents who had already matched to their R4 program.

Thus, our recruitment pool consisted of the 18 fourth year Internal Medicine residents (i.e. first year subspecialty fellows, including those in a General Internal Medicine fellowship) and 19 third year Internal Medicine residents at the University of Alberta who had already chosen their subspecialty. All residents were recruited via email and/or letters. Information sheets were provided both with the recruitment email/letter and at the focus group. Adequate time was set aside for questions or concerns about the study before each focus group session began.

### Data collection

The same person [V.J.D.] conducted each focus group discussion using a guide developed by the two authors. We used the interview guide approach [14] in which the two authors specified ahead of time the topics to be covered, but we were not rigid in the exact wording and sequence of the questions. Thus, the focus group discussions remained to some extent conversational. The discussions ran between 60-90 minutes and began with open-ended questions such as "Why did you choose to pursue the subspecialty to which you matched?" and "How did you go about making your decision to pursue this fellowship?" After an open discussion, the discussion shifted to close-ended question such as "Did any of the following factors affect your decision?" with a list of factors pulled from previous studies and those the authors felt might be important (see Table 1).

**Table 1:** Focus group questions

Open-Ended Questions
Why did you choose to pursue the subspecialty to which you matched?
How did you go about making your decision to pursue this fellowship?
When did you decide on this subspecialty?
Was there anything in particular during your residency that caused you to pick this fellowship over another?

**Closed Ended Questions**

Did any of the following affect your decision?
*Items drawn from literature:*
-projected income when training completed^2,9^
-lifestyle (time for non-work activities and/or family)^2,9^
-subject matter^2^
-ability to do procedures/technical skills^2^
-breadth of practice^2^
-long-term vs. short-term relationships with patients^2^
-inpatient vs. outpatient care^2^
-role models^2^
-negative influences ("why would you want to pursue that?")^2^
-marital status^9^
-personal debt^9^
-wanted or did not want to deal with certain patient populations^9^
*Items postulated to be important by authors:*
-opportunities for teaching
-opportunities for research
-prestige/respect from colleagues
-prestige/respect from those outside medicine (friends/family)
-job prospects/opportunities/openings
-national trends
-length of training
-other personal issues

We conducted three focus groups. Our goal for each focus group was to have six to eight residents participate [14]. The first focus group consisted of seven fourth year residents (participants A-G); the second involved seven third year residents after they had matched to their fellowship (participants H-N); and the third involved five residents (originally six but one resident was unable to attend at the last minute), some third and some fourth year (participants O-S). Nine of the participants were male and ten were female. Residents had matched to a wide range of subspecialties: Cardiology, Endocrinology, Gastroenterology, General Internal Medicine, Hematology, Infectious Diseases, Nephrology, Oncology, Respirology and Rheumatology. The responders were a fairly representative sample of the residents given all the different subspecialties were represented.

### Data analysis

All focus group discussions were tape recorded and fully transcribed. Trustworthiness was ensured with interrater reliability, triangulation, and member checking. For interrater reliability, the two investigators (V.J.D., N.K.) reviewed transcripts independently and performed an inductive content analysis to discover patterns, themes and categories [15]. At least 80% agreement of themes was reached and then any discrepancies in themes and use of supporting participant comments were resolved by consensus. All of the themes were identified in the data from the open-ended questions. Triangulation included data triangulation with both third and fourth year residents and was carried out at three different times during an academic year, and investigator triangulation as one investigator (V.J.D) was a resident at the time and the other (N.K.) was a practicing physician.

Member checking was carried out after data analysis by contacting a subset of our participants with at least one representative from each of the three focus groups. All of these participants felt the results and themes were accurate representations of their views.

## Results

**Figure 1:**
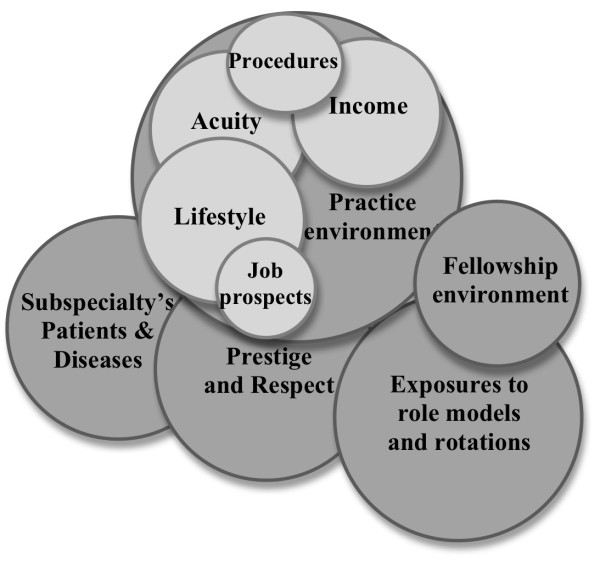
**Visual representation of number of comments for each theme**.

We identified five major themes regarding reasons why residents chose their fellowship program. Saturation of themes was reached by the third focus group. See Table 2 for the number of comments and respondents associated with each theme. See Figure 1 for a visual representation of the number of comments for each theme. See Additional File 1 for more representative quotes. Themes are arranged in descending order of number of attributable comments.

**Table 2:** Comments and respondents for each theme

Theme	# of comments	# of different respondents
Practice environment	66	18

-lifestyle	19	14

-acuity	17	12

-income	15	7

-procedures	8	8

-job	7	5

Exposure in rotations and to role models	42	16

Subject Matter	34	16

Prestige/Respect	34	10

Fellowship	19	10

### Theme 1 - Practice environment including acuity of practice, ability to do procedures, lifestyle, job prospects and income

Residents were fairly specific about their desires of an outpatient, inpatient or combined practice.

E (Male): "I'd like to do a combination [of inpatient and outpatient]. It's like variety, I get too bored doing one thing."

As expected many residents cited lifestyle as being important.

J (Female): "I am in a later phase of my life so ... lifestyle is another thing I want to have and I don't want to have too many calls because being an internist you have to be on call."

Job prospects were a significant factor for some but not all.

A (Male): "I don't think the job market in Canada is good at this moment ... but honestly I don't think of that at all."

Income appeared to be important to some, but not others.

C (Male): "I will never be out of debt. I will die in debt. So it really doesn't matter. I'm so bad with money. Money didn't play a factor into it, it honestly didn't because we'll make a princely sum no matter what area of medicine we go into compared to the rest of the world."

R (Male): "Some subspecialties pay twice as much so I could work half as much and make the same amount. That's pretty cool! So you know that's definitely a factor. The remuneration, definitely part of it."

S (Female): "Like who hasn't heard that when you do a Nephro consult or a GI consult you get paid like what is it 50 or 75 bucks more than when you do an Internal Medicine consult and you only answer you know one question versus doing the whole system review and being more thorough."

### Theme 2 - Exposure in rotations and to role models

Both positive and negative experiences on rotations had a significant impact for residents.

N (Female): "I think it's made or broken by the mentorship that you receive and the influences that you've [had] ... especially through the first two years of residency."

L (Female): "Seeing someone happy in their practice and in their life helps you visualize what your practice in life could be like."

### Theme 3 - Interest in subspecialty's patient population and common diseases (including breadth)

Residents felt a subspecialty's common diseases and/or patient populations were important in making the decision.

E (Male): "...every subspecialty has its bread and butter... I think you have to be willing and able to deal with those diseases... For example, pulmonary although interesting, I find I hate COPD..."

K (Male): "Last few years, medicine has been ... getting away from good General Internal Medicine to becoming more toward Geriatrics."

### Theme 4 - Prestige and respect

Most residents felt prestige and respect for their chosen specialty was important.

S (Female): "Well I mean if you're going to spend a decade in post-secondary education you want to come out as someone who's competent, respected and contributing."

M (Male): "I was driven away from [General] Internal Medicine because I think Internists are not respected here and they're forced to do work that is not related to their specialty."

There was actually a lot of discussion about how General Internal Medicine was perceived as a dumping ground for patients nobody else wanted and this was cited as a large deterrent away from pursuing General Internal Medicine.

### Theme 5 - Fellowship training environment including fellowship program resources and length of training

Residents also considered the perceived strength of a program and its resources.

D (Female): "...I was choosing between a subspecialty and general internal medicine and I think the factors that pushed towards the subspecialty was partly the fact that I would like to do some critical care in a smaller community and I felt that the subspecialty would prepare me better because I would get more ICU training, I would get more procedural training and more physiology training..."

R (Male): "in the General Internal Medicine program even as a fellow, you're at the bottom rung for booking clinics. Medical students get priority over you for booking clinics."

Most residents were prepared to do two years of fellowship training after their core three years of Internal Medicine but not always more.

D (Female): "It would have deterred me from doing something that would have been longer than two years... in something like Cardiology... you'll be an R8 still doing your echo fellowship or whatever... I'm ready to be done."

With a recent push in Canada to promote a fifth year of training in General Internal Medicine, residents are questioning what the advantages are.

K (Male): "...to stay in a bigger city... I have to spend fifth year of training [in General Internal Medicine] and I have seen people do that just to have extra niche... So if you end up spending five years and only getting one certificate as compared to getting two certificates in subspecialty, ... that opens up your boundaries a lot."

R (Male): "Yeah. We're all going to be general internists. So what would be the point of doing the General Internal Medicine [fellowship]?"

As you can see, some residents interested in General Internal Medicine looked at their desired career (for example, a career that included a subspecialty-based procedure) to see if they could achieve what they needed in a General Internal Medicine fellowship. Many felt it made more sense to take on subspecialty training to ensure they received the desired training and then they could practice General Internal Medicine in addition to their subspecialty. This largely stemmed from a perceived inability of a General Internal Medicine fellowship to provide the necessary training, and thus this was not a deterrent from practicing General Internal Medicine, but from pursuing a General Internal Medicine fellowship.

## Discussion

To summarize, the factors that our residents appear to consider in the R4 match can be categorized into five themes: 1) Practice environment including acuity of practice, ability to do procedures, lifestyle, job prospects and income; 2) Exposure in rotations and to role models; 3) Interest in a subspecialty's patient population and common diseases; 4) Prestige and respect of subspecialty; 5) Fellowship training environment including fellowship program resources and length of training. The primary goal of our study was to determine factors that contributed to residents' decision in the R4 subspecialty match, and then we could infer why residents were not pursuing less sought after subspecialties such as General Internal Medicine.

As mentioned previously, most of the work in this area has been done on American trainees. However in Canada, General Internal Medicine is a very different specialty. Becoming a general internist in the United States does not require additional training beyond three years of residency, although some residents pursue 1-2 year fellowships in General Internal Medicine. In Canada, the training involves three years of a core Internal Medicine residency and then an additional required one or two years in a General Internal Medicine fellowship program depending on the university and the desires of the fellow. Hence, the length of training of a general internist is often the same as that of a subspecialist. This results in a different role of the Canadian general internist. Unlike in the United States where a general internist is often an outpatient-based primary care physician, in Canada the general internist acts as a consultant for primary care physicians for a wide variety of medical conditions. Thus the Canadian general internist more often sees patients with multisystem diseases and undifferentiated problems compared to his/her American counterpart [16]. Given the difference in roles between the countries, it would not be surprising if residents had different reasons for pursuing General Internal Medicine as a specialty/career in Canada.

Horn and colleagues [11] are the only investigators whom we are aware of that have carried out similar work in Canadian Internal Medicine residents. They identified four themes: 1) mentorship, role models and experience on rotations; 2) patients, practice type and personal fit; 3) lifestyle and family; and 4) future job opportunities and finances [11]. It is reassuring to see we also identified these themes, but we also identified two new themes for Canadian residents. The first is the perceived lack of prestige and respect for some subspecialties with General Internal Medicine being the most cited example. The second is the fellowship training environment which includes two new factors, the resources available to a fellowship program, be it clinic time or access to procedures, and length of training, specifically the recent push towards a two-year General Internal Medicine fellowship as a possible deterrent.

The general consensus amongst residents is that if you now have to train for two years in a General Internal Medicine fellowship, but you can practice General Internal Medicine as a subspecialist, why not do a subspecialty? *Participant R (Male): "Yeah. We're all going to be general internists. So what would be the point of doing the General Internal Medicine [fellowship]?" *A study by Thomas and colleagues [17] demonstrated that the majority of American internal medicine residents feel that three years is the adequate training time for a general internist. In Canada our current training is four to five years with a recent move to standardize training across the country at five years. There is no equivalent Canadian study to Thomas et al. [17] but our qualitative data suggests this increase will be a deterrent.

We need to address the ongoing discrepancy between societal need and the output of our training programs, a problem that as mentioned earlier is a global one. We found practice environment to be possibly the most important factor, but there are some misconceptions about this. First, most residents only see the practice of an academic physician, which is far different than a community physician's practice. Thus training programs need to continue increasing the exposure of residents to community settings. Second, we have a problem with our residents' perceptions of remuneration. Locally we have had significant changes to the remuneration of General Internal Medicine specialists that reflects the complexity of the work they do. For the average complex consult in many Canadian provinces, a general internist bills more than a subspecialist. However, our residents are unaware of this: *Participant S (Female): "Like who hasn't heard that when you do a Nephro consult or a GI consult you get paid like what is it 50 or 75 bucks more than when you do an Internal Medicine consult and you only answer you know one question versus doing the whole system review and being more thorough." *Hence we need to address any misconceptions.

As for our new themes, prestige and respect of a specialty are difficult to change. However the resources of a training program are not. If the need is for General Internal Medicine specialists, we feel organizations need to lobby those who set the fee schedules for fairer remuneration and to lobby universities to give priority to these training programs and not, for example, have the General Internal Medicine fellow in a queue behind medical students for booking clinics for their training.

## Limitations and Future Directions

The results of this study need to be interpreted with the following two limitations in mind. First, the trustworthiness of the data could have been improved. Although we did have an initial interrater reliability (> 80%) based on verbal discussion, we did not calculate the exact interrater reliability for themes and the comments assigned to themes. We did have some data and investigator triangulation, but these could have been improved by using practicing physicians in addition to residents as our participants, and by having a third investigator with a different perspective. In addition, we could have used method triangulation with multiple methods in addition to focus group discussions such as interviews and questionnaires. Having an observer during the focus group discussions to record non-verbal behavior would also be an improvement.

Second, a total of 19 residents from a single Canadian institution participated in this research and this number may be considered small and perhaps not generalizable. At the institution there were a total of 37 third and fourth year residents at the time of the study of which we were able to recruit just over 50%. These 19 residents represented all of the possible subspecialties with a good mix of gender, thus allowing us to feel that they were representative of the larger group. Perhaps most importantly, we achieved saturation of factors with the third focus group suggesting that the 19 participants covered all relevant factors. We should point out that the third focus group, which was necessary for saturation, was heterogeneous in that it consisted of both third and fourth year residents. This, however, was a product of the number of residents available to participate at the time and should not undermine the importance of reaching saturation.

The intention of this study was to identify factors that Internal Medicine residents might consider when choosing their fellowship and career. Although we have been able to ascertain the relative importance of these factors to some degree (see Table 2 and Figure 1), the next step would be to use these factors to design a survey using a larger sample size at multiple institutions to add to the research in this field.

## Conclusion

A variety of factors contributing to Internal Medicine residents' fellowship choice have been identified in previous studies. This study makes an important contribution to the literature in that it identifies new factors that Canadian residents consider when pursuing a fellowship including prestige and respect of a subspecialty, resources of the fellowship program, and concern about the recent trend in Canada towards a two-year General Internal Medicine fellowship. We also offer some thoughts as to how to address this gap between training program output and societal need. Future surveys that incorporate these factors would continue to add to our understanding of residents' subspecialty choices in Internal Medicine.

## Competing interests

The authors declare that they have no competing interests.

## Authors' contributions

All authors listed have contributed sufficiently to the project to be included as authors, and all those who are qualified to be authors are listed in the author byline. NK conceived the original idea for the project. VJD worked on the design of the project with the input of NK. VJD carried out all of the focus group discussions. VJD and NK analyzed the data. VJD drafted the first version and subsequent revisions of the manuscript; NK reviewed and edited the various versions of the manuscript. Both authors approved the final draft.

## Pre-publication history

The pre-publication history for this paper can be accessed here:

http://www.biomedcentral.com/1472-6920/11/44/prepub

## Supplementary Material

Additional file 1 **Representative quotes**. Additional file with representative quotes. Click here for file
