# Supplementary material for: Determinants of internal medicine residents' choice in the canadian R4 Fellowship Match: A qualitative study
Source: BMC Med Educ. 2011 Jun 29;11:44. doi: 10.1186/1472-6920-11-44 (PMC3146947; doi:10.1186/1472-6920-11-44)
Supplement: Additional file 1 — Representative quotes. Additional file with representative quotes. [file 1472-6920-11-44-S1.PDF]

Additional File 1 – Representative quotes

Theme 1 – Practice environment including acuity of practice, ability to do procedures, lifestyle, job prospects and income

E (Male): "I'd like to do a combination [of inpatient and outpatient]. It's like variety, I get too bored doing one thing."

D (Female): "I wanted to do some acute care and inpatient medicine so I wouldn't have chosen a specialty that was mainly clinic based."

K (Male): "…it allows me to do some procedures which I will never be able to do in General Internal Medicine."

O (Female): "It was something that I sacrificed because I really liked doing procedures but then I realized … with procedures … comes acute care which I liked but which takes a toll on your lifestyle."

C (Male): "I mean lifestyle is certainly an important factor and it made me rule out things that I really liked in Internal – like Intensive Care."

J (Female): "I am in a later phase of my life so … lifestyle is another thing I want to have and I don't want to have too many calls because being an internist you have to be on call."

F (Female): "For me, I wanted to … be in a field that was in demand where I could make my own hours … rather than having a really competitive field that maybe dictated your own hours…"

A (Male): I don't think the job market in Canada is good at this moment … but honestly I don't think of that at all

C (Male): “I will never be out of debt. I will die in debt. So it really doesn’t matter. I’m so bad with money. Money didn’t play a factor into it, it honestly didn’t because we’ll make a princely sum no matter what area of medicine we go into compared to the rest of the world.”

G (Male): “my specialty has lots of money attached to it, but I’d probably do it for a lot less, I mean I know that there’s a huge income discrepancy between [my specialty] and everyone else.”

R (Male): “Some subspecialties pay twice as much so I could work half as much and make the same amount. That’s pretty cool! So you know that’s definitely a factor. The remuneration, definitely part of it.”

S (Female): “Like who hasn’t heard that when you do a Nephro consult or a GI consult you get paid 50 or 75 bucks more than when you do an Internal Medicine consult and you only answer one question versus doing the whole system review and being more thorough. Right?”

<u>Theme 2 – Exposure in rotations and to role models</u>

D (Female): “One was a really positive experience on the consult service where I really thought ‘this is really interesting’. I had not thought of this [yet] as a career but I really loved it.”

I (Male): If there was anything that could change my mind would be just getting more clinic in R1 and R2 years for Internal Medicine rather than working as hospitalists.

N (Female): “I think it’s made or broken by the mentorship that you receive and the influences that you’ve [had] … especially through the first two years of residency.”

L (Female): “Seeing someone happy in their practice and in their life helps you visualize what your practice in life could be like.”

Theme 3 – Interest in subspecialty's patient population and common diseases (including breadth)

E (Male): "…every subspecialty has its bread and butter… I think you have to be willing and able to deal with those diseases… For example, pulmonary although interesting, I find I hate COPD…"

N (Female): "I kind of made the decision… based on …what kind of patients did I get all excited about when I get a call from emerg… there were certain types of consults that I would be like 'Oh, this sounds really exciting!'"

C (Male): "…I find HIV fascinating and … I was admitting really interesting HIV patients [which] kind of cemented things for me."

K (Male): "Last few years, medicine has been … getting away from good General Internal Medicine to becoming more toward Geriatrics."

R (Male): "I prefer to be an expert in my area and feel I really know what's going on in my area at all times, all research. It's much easier for subspecializing to keep up on top of that. I can't imagine how I would do it in General Internal Medicine. It's daunting."

S (Female): "it takes a lot of guts to see people that are that sick and that complex in so many systems"

Theme 4 – Prestige and respect

S (Female): "Well I mean if you're going to spend a decade in post-secondary education you want to come out as someone who's competent, respected and contributing."

M (Male): "I was driven away from [General] Internal Medicine because I think Internists are not respected here and they're forced to do work that is not related to their specialty."

K (Male): "prestige was an important thing 'cause you could hear politics of dumping patients to [General Internal] medicine… if I am doing something I want to be valued in what I'm doing."

Theme 5 – Fellowship training environment including fellowship program resources and length of training

S (Female): "I had two subspecialties because one kind of came up late … one I was considering here and one I was considering in another city based on the strength of the program … I think ultimately what decided it was … it's better funded than the other"

D (Female): "…I was choosing between a subspecialty and general internal medicine and I think the factors that pushed towards the subspecialty was partly the fact that I would like to do some critical care in a smaller community and I felt that the subspecialty would prepare me better because I would get more ICU training, I would get more procedural training and more physiology training…"

D (Female): "[Length of training] would have deterred me from doing something that would have been longer than two years… in something like Cardiology… you'll be an R8 still doing your echo fellowship or whatever… I'm ready to be done."

S (Female): “I know that if I want to go academic I have to do a lot more [than two years] and I don’t know whether I’m prepared to do a lot more.”

K (Male): “…to stay in a bigger city… I have to spend fifth year of training [in General Internal Medicine] and I have seen people do that just to have extra niche… So if you end up spending five years and only getting one certificate as compared to getting two certificates in subspecialty, that’s more lucrative, that open up your boundaries a lot.”

D (Female): “…instead of doing a fourth year general medicine and adding on another fifth year to learn procedures etcetera, etcetera, why not just do subspecialty and you can still practice general medicine as a subspecialist and yet you get all the benefits of the extra training and you write the extra exam.”
